# Agreement between PSMA-RADS and E-PSMA systems in classifying [^18^F]PSMA-1007 PET/CT lesions among prostate cancer patients: exploring the correlation between lesion size and uptake

**DOI:** 10.3389/fmed.2024.1368093

**Published:** 2024-03-12

**Authors:** Miguel Mendoza-Ávila, Hiram Esparza-Pérez, Juan Andrés Castillo-López, Edel Rafael Rodea-Montero

**Affiliations:** ^1^Department of Radiology, Hospital Regional de Alta Especialidad del Bajío, León, Mexico; ^2^Faculty of Medicine, Universidad Nacional Autónoma de México, Ciudad de México, Mexico; ^3^Department of Nuclear Medicine, Hospital Regional de Alta Especialidad del Bajío, León, Mexico; ^4^Department of Research, Hospital Regional de Alta Especialidad del Bajío, León, Mexico; ^5^UPIIG, Instituto Politécnico Nacional, Silao de la Victoria, Mexico

**Keywords:** [^18^F]PSMA-1007, lesion size, PET/CT, prostate cancer, SUVmax

## Abstract

**Purpose:**

To determine the agreement between the PSMA-RADS and E-PSMA standardized reporting systems in the classification of [^18^F]PSMA-1007–uptaking lesions identified on PET/CT scan in patients with prostate cancer (PCa) and post-prostatectomy with suspected recurrent disease (local recurrence, regional nodal involvement and distant metastases), based on biochemical recurrence, while also exploring the correlation between lesion size and tracer uptake.

**Materials and methods:**

A retrospective cross-sectional study of 32 post-prostatectomy PCa patients who had suspected recurrent disease based on biochemical recurrence post-prostatectomy (prostate-specific antigen values that are 0.2 ng/mL or higher) underwent [^18^F]PSMA-1007 PET/CT scan. The recurrent disease PCa lesions were characterized and subsequently classified using two standardized reporting systems (PSMA-RADS and E-PSMA). The lesions were grouped based on anatomical site, their size and SUVmax were compared using Kruskal-Wallis test with Dunn-Bonferroni *post hoc* tests. Spearman correlation coefficients were calculated between the size of the lesions and their SUVmax of the radiotracer [^18^F]PSMA-1007 for all the lesions and when grouped by anatomical site. Additionally, the agreement between lesion classifications was assessed using Cohen’s kappa index.

**Results:**

Only 32 (69.98 ± 8.27, men) patients met the inclusion criteria, a total of 149 lesions with avid uptake of [^18^F]PSMA-1007 were identified. Positive correlation (*r* = 0.516, *p* < 0.001) was observed between the size of the metastatic prostate cancer lymph node lesions and their [^18^F]PSMA-1007 uptake. Substantial agreement was noted between the PSMA-RADS and E-PSMA classification system scores among all lesions (κ = 0.70, *p* < 0.001), with notable discrepancies primarily among lymph node lesions.

**Conclusion:**

Our findings revealed a positive correlation between the size of the metastatic prostate cancer lymph node lesions and [^18^F]PSMA-1007 uptake, and although there was substantial agreement between the PSMA-RADS and E-PSMA classification systems, there were discrepancies mainly among the lymph node lesions.

## 1 Introduction

Prostate cancer (PCa) is the most common type of cancer in men, with an incidence of one in six in developed countries (1). Prostate-specific membrane antigen (PSMA) is a type II transmembrane glycoprotein that is used as a radiopharmaceutical in positron-emission tomography/computed tomography (PET/CT) scans (2, 3). It can be expressed in various anatomical sites, such as the salivary glands, renal tubules, glial cells of the central nervous system, the small intestine and the prostate gland, where high levels of PSMA are expressed in the presence of PCa (4–6). The extracellular domain of PSMA has a high affinity for urea-based ligands (7), which has led to the development of various ligands labeled with radioisotopes for diagnostic purposes, such as gallium-68 [^68^Ga] and fluorine-18 [^18^F], and for theranostic purposes, such as lutetium-177 [^177^Lu] (8, 9).

Currently, PSMA PET/CT in combination with various agents constitute one of the best imaging diagnostic methods for patients with a high or very high risk of PCa with suspected metastasis (10). In particular, [^18^F] labeled PSMA has demonstrated safety, expected tissue biodistribution, and significant PCa-specific uptake (11). Evidence shows a detection rate for PCa with biochemical recurrence of up to 86%, with a sensitivity of 61.4% and a specificity of 88.3% for [^18^F]PSMA PET/CT (12, 13). Additionally, [^18^F]PSMA-1007 generates images with higher spatial resolution than [^68^Ga]Ga-PSMA-11 and [^18^F]PSMA-DCFPyL, improving the tumor-to-background ratio, increasing the radiopharmaceutical decay half-life (110 min), and achieving better lesion delineation at the pelvic level (14).

Importantly PSMA PET/CT yields a semiquantitative measurement of the standardized uptake value (SUV) of the radiotracer, which is useful in oncology for detecting primary or metastatic sites of PCa, differentiating between benign and malignant tumors, comparing lesions seen on CT scans, planning treatment, monitoring the response to treatment and/or detecting recurrent disease (15). Standardized reporting systems, particularly the PSMA-RADS (16) proposed by the American College of Radiology or the E-PSMA (17) supported by the European Association of Nuclear Medicine, were used to describe these results.

The aim of this study was to determine the agreement between the PSMA-RADS and E-PSMA standardized reporting systems in the classification of [^18^F]PSMA-1007–uptaking lesions identified on PET/CT scans in patients with PCa and post-prostatectomy with suspected recurrent disease (local recurrence, regional nodal involvement and distant metastases), based on biochemical recurrence, while also exploring the correlation between lesion size and tracer uptake.

## 2 Materials and methods

### 2.1 Patients

This study included a total of 32 patients with a history of PCa and prostatectomy, who had suspected recurrent disease (local recurrence, regional nodal involvement and distant metastases), based on biochemical recurrence (BCR) post prostatectomy that is defined as at least two prostate-specific antigen (PSA) values that are 0.2 ng/mL or higher (18, 19). Patient selection was conducted retrospectively, employing a consecutive sampling approach based on the availability of individuals who underwent [^18^F]PSMA-1007 PET/CT scans at the Department of Nuclear Medicine within a tertiary care hospital between January 1, 2021, and December 31, 2022. The inclusion criteria were male sex ≥18 years, post-prostatectomy PCa, and data from noncontrast [^18^F]PSMA-1007 PET/CT scan performed from the vertex of the calvaria to the middle third of the femur at a 120 kV voltage for suspected recurrent disease based on BCR. The exclusion criteria were noncontrast PET/CT images with the presence of movement artifact, treatment with diuretics at the time of the PET/CT scan, and the presence of kidney and/or liver disease.

Data, including clinical (age, weight, height, body mass index), biochemical (PSA at diagnosis of PCa, PSA nadir, and PSA at the time of PET/CT scan), histopathological [pathological TNM grade, Gleason score, clinical risk stratification according to the National Comprehensive Cancer Network (NCCN) system], surgical approach to prostatectomy, and additional treatment received (endocrinological, radiotherapeutic, chemotherapeutic) prior to PET/CT scan, were obtained from the medical records of the patients. At the time of the PET/CT scan, the patients were receiving care from the Urology Oncology Service.

### 2.2 Image acquisition

The PET/CT scans were performed with a 16-slice Biograph mCT scanner (Siemens Healthineers AG, Erlangen, Germany). All patients received a weight-based dose of [^18^F]PSMA-1007 of 4 MBq (0.11 mCi) per kg. Images were acquired at 90 min without intravenous iodinated contrast medium from the vertex of the calvaria to the middle third of the femur after intravenous application of the PSMA radiotracer. The PET data were acquired using the time-of-flight function with two iterations and 21 subsets. The CT portion of the PET/CT scan was acquired with a pitch of 1.2 mm, automatic mA, 120 kV, a rotation time of 0.5 s, and a slice thickness of 5 mm. All PET/CT scans were taken with the same PET/CT system, which was assessed daily with a Germanium 68 (Ge-68) source for quality control during the study period.

### 2.3 Image analysis

The images acquired from the [^18^F]PSMA-1007 PET/CT scans were retrospectively accessed through the picture archiving and communication system (PACS). In collaboration, a certified nuclear medicine physician and a certified radiologist, each possessing 3 years of experience in interpreting PSMA PET/CT scans and proficient in utilizing the PSMA-RADS and E-PSMA reporting systems, identified and characterized every distinct well-marginated lesion in regard to its precise location and morphology. Notably, this process was conducted independent of any clinical patient details.

**Figure 1:**
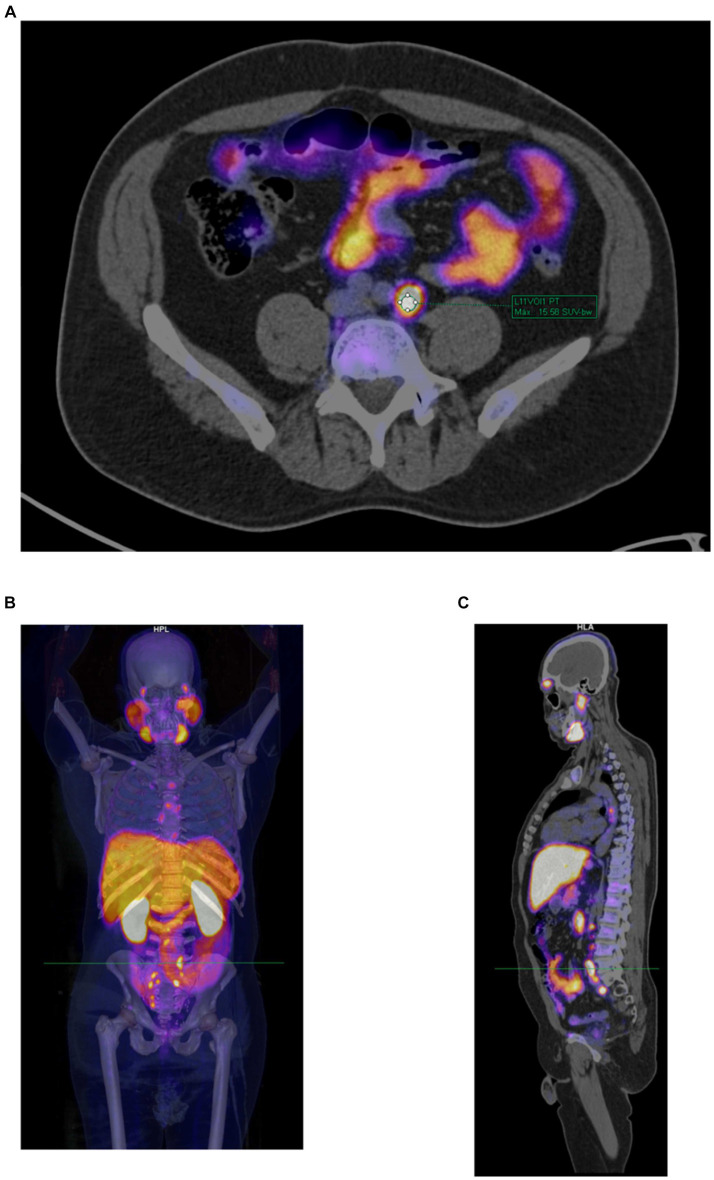
Example of a PET/CT scan with [^18^F]PSMA-1007 in a 53-year-old patient with prostate cancer and post-prostatectomy who had suspected recurrent disease. **(A)** Manual determination of [^18^F]PSMA-1007 uptake in a lymph node according to the semiquantitative SUVmax (=15.58) using the volume of interest (VOI) tool. **(B)** Volumetric reconstruction used to show the anatomical site of the evaluated lesion. **(C)** Sagittal section showing the anatomical site of the evaluated lesion.

Additionally, measurements of size (diameter on the short axis in mm), SUVmax, and reference SUVmax values for each patient (in the blood pool, spleen, and parotid gland) were conducted to assess the molecular imaging PSMA (miPSMA) expression of each lesion. These measurements were performed by the radiologist under the supervision of the nuclear medicine physician. Figure 1 shows an example of the SUVmax evaluation of a lesion.

### 2.4 Lesion classification

The lesions were classified by the nuclear medicine physician using two standardized reporting systems: the PSMA-RADS (16) proposed by the ACR and its European equivalent, the E-PSMA system (17) based on the Prostate Cancer Molecular Imaging Standardized Evaluation (PROMISE V2) (20). Both systems classify lesions into five categories, the definitions of which are detailed in Table 1.

**Table 1:** Definitions of the PSMA-RADS and E-PSMA prostate cancer lesion classification systems.

Classification system				
PSMA-RADS			E-PSMA	
Score		Definition	Score	Definition
PSMA-RADS-1	PSMA-RADS-1A	Lesions without radiotracer uptake that are definitively benign	E-PSMA-1	Benign lesion without abnormal PSMA uptake
	PSMA-RADS-1B	Lesions with radiotracer uptake that are definitively benign		
PSMA-RADS-2		Likely benign: Low level radiotracer uptake in bone or soft tissue sites that would be atypical for metastatic prostate cancer	E-PSMA-2	Most likely, benign lesion: faint PSMA uptake (equal or lower than background) in a site atypical for prostate cancer
PSMA-RADS-3*	PSMA-RADS-3A	Equivocal radiotracer uptake in soft tissue lesions such as lymph nodes in a distribution typical for prostate cancer	E-PSMA-3	Equivocal finding: faint uptake in a site typical for prostate cancer or intense uptake in a site atypical for prostate cancer
	PSMA-RADS-3B	Equivocal radiotracer uptake in bone lesions that are not clearly benign		
	PSMA-RADS-3C	Lesions that would be atypical for prostate cancer but have high levels of uptake and many represent a nonprostate malignancy		
	PSMA-RADS-3D	Lesions that are concerning for the presence of prostate cancer or a nonprostate malignancy but lack radiotracer uptake		
PSMA-RADS-4		Likely prostate cancer: lesions with high radiotracer uptake that would be typical for prostate cancer but lack a definitive anatomic abnormality	E-PSMA-4	Most likely, prostate cancer: intense uptake in typical site of prostate cancer, but without definitive findings on CT **
PSMA-RADS-5		Definitively prostate cancer: lesions with high levels of radiotracer uptake and corresponding anatomic findings that are indicative of the presence of prostate cancer	E-PSMA-5	Definitive evidence of prostate cancer: intense uptake in a typical site of prostate cancer, with definitive findings on CT

*Further work-up can be considered. **A definitive finding on CT means the presence of a real anatomical substrate on the CT.

### 2.5 Statistical analysis

All the data were analyzed using the statistical software R version 3.6.0 (21). Missing values for the variables determined in the study were not imputed, and patients were not included in the analysis for those variables. Descriptive statistics were determined for the characteristics of the patients and their lesions. The statistical analysis of lesion size and SUVmax variables were performed using non-parametric methods due to the unmet assumptions for parametric statistics, as indicated by the Kolmogorov–Smirnov test results (*p* < 0.001 in both cases). The lesions were grouped based on their anatomical site (surgical bed, lymph nodes, bone, locoregional spread, and other sites). The lesion sizes and SUVmax were subjected to comparison using the Kruskal-Wallis test, with subsequent utilization of the Dunn-Bonferroni *post-hoc* tests, where appropriate. Spearman correlation coefficients were calculated between the size of the lesions and the SUVmax of the radiotracer [^18^F]PSMA-1007 for all the lesions and when grouped by anatomical site. In addition, the agreement between lesion classifications (PSMA-RADS and E-PSMA) was assessed using Cohen’s kappa index. To assess the strength of agreement, the Landis and Koch assessment was used (22) (< 0.00 poor, 0.00–0.20 slight, 0.21–0.40 fair, 0.41–0.60 moderate, 0.61–0.80 substantial, 0.81–1.00 almost perfect). A significance level of α = 0.05 was used for all tests.

## 3 Results

In the retrospective analysis, a total of 32 postoperative PCa patients who underwent [^18^F]PSMA-1007 PET/CT were included. The mean (± SD) age of the patients was 69.98 ± 8.27 years (range 53.27–86.32 years). The characteristics of the patients are detailed in Table 2. All patients received care from the Urology Oncology Service, and [^18^F]PSMA-1007 PET/CT scans were performed because of BCR. No patient met the exclusion criteria, which included noncontrast PET/CT images showing movement artifacts, the presence of diuretic treatment during the PET/CT scan, and kidney or liver disease.

**Table 2:** Patient characteristics (*n* = 32).

	Mean (SD)	Median (IQR)	
Age (years)	69.98 (8.27)	68.72 (65.79–75.28)	
Weight (kg)	70.13 (14.56)	73.25 (58.50–81.25)	
Height (m)	1.63 (0.06)	1.64 (1.60–1.69)	
BMI (kg/m2)	26.2 (4.57)	27.34 (22.25–30.27)	
Malnourished (BMI < 18.5), *n* (%)	1 (3.13%)		
Normal weight (18.5 ≤ BMI < 25), *n* (%)	11 (34.38%)		
Overweight (25 ≤ BMI < 30), *n* (%)	11 (34.38%)		
Obese (BMI ≥ 30), *n* (%)	9 (28.13%)		
PSA			
At the time of diagnosis (ng/ml)	618.08 (1612.47)	60.00 (23.29–168.00)	*n* = 29
Nadir (ng/ml)	1.74 (3.55)	0.18 (0.06–1.93)	*n* = 27
At the time of [^18^F]PSMA-1007 PET/CT scan (ng/ml)	27.15 (57.65)	3.31 (0.69–14.03)	
Pathological TNM stage			
T2, *n* (%)	19 (63.3%)		*n* = 30
T3, *n* (%)	7 (23.3%)		
T4, *n* (%)	4 (13.3%)		
N0, *n* (%)	16 (57.1%)		*n* = 28
N1, *n* (%)	12 (42.9%)		
M0, *n* (%)	11 (36.7%)		*n* = 30
M1, *n* (%)	19 (63.3%)		
Gleason score			
6, *n* (%)	3 (10%)		*n* = 30
7, *n* (%)	6 (20%)		
8, *n* (%)	9 (30%)		
9, *n* (%)	12 (40%)		
Risk stratification for localized prostate cancer (NCCN)			
Low, *n* (%)	2 (6.3%)		
Intermediate, *n* (%)	6 (18.8%)		
High, *n* (%)	6 (18.8%)		
Very High, *n* (%)	18 (56.3%)		
Surgical approach			
TURP, *n* (%)	15 (46.9%)		
TURP and SBO, *n* (%)	7 (21.9%)		
RP, *n* (%)	9 (28.1%)		
RP and SBO, *n* (%)	1 (3.1%)		
Additional treatment			
Endocrinological, *n* (%)	31 (96.9%)		
Radiotherapy, *n* (%)	17 (53.1%)		
Chemotherapy, *n* (%)	7 (21.9%)		
Number of lesions with avid uptake of [^18^F]PSMA-1007	4.66 (3.49)	3.50 (1.00–7.50)	

Unless otherwise indicated, all values are given by the mean (standard deviation) and median (interquartile range). BMI: body mass index. PSA: prostatic-specific antigen. NCCN: National Comprehensive Cancer Network. TURP: transurethral resection of the prostate. SBO: simple bilateral orchiectomy. RP: open radical prostatectomy.

**Figure 2:**
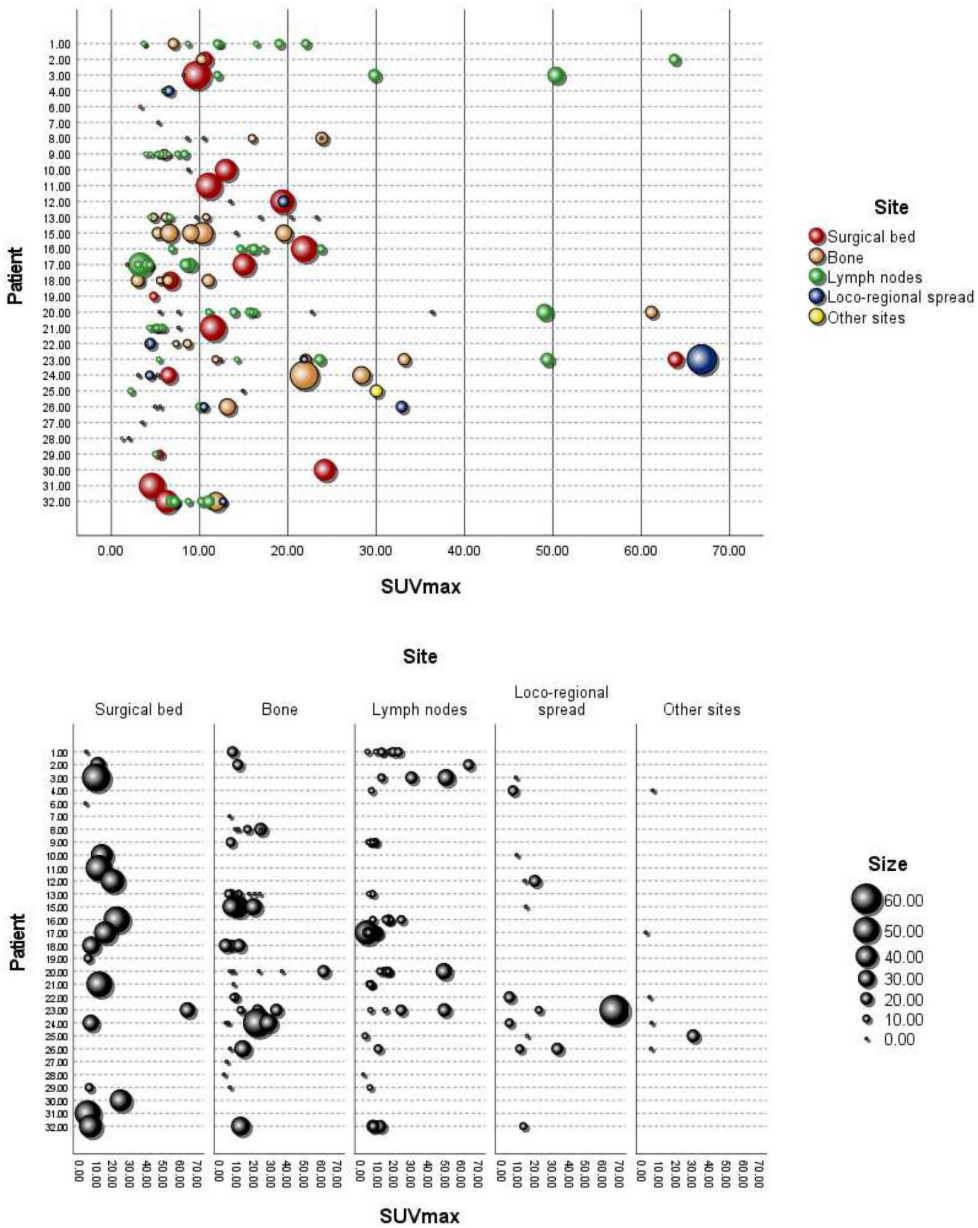
[^18^F]PSMA-1007 uptake (SUVmax) in lesions per patient, with the size in mm and grouping by anatomical site. Loco-regional spread: bladder, seminal vesicle, and rectum. Other sites: thyroid, kidney, and bone marrow.

In addition, we considered a total of *n* = 149 lesions with avid uptake of [^18^F]PSMA-1007; the mean (± SD) number of lesions per patient was 4.66 (± 3.49, range 0–11). Figure 2 shows the [^18^F]PSMA-1007 uptake (SUVmax) of lesions per patient, with the size of the lesions depicted in mm, grouped by anatomical site. The distribution of the avid [^18^F]PSMA-1007 uptake sites and the characteristics (size and SUVmax) of the lesions are detailed in Table 3. Notably, of the 32 patients analyzed, 2/32 (6.25%) did not present with lesions with avid [^18^F]PSMA-1007 uptake. The largest metastatic PCa lesions, in terms of size, were found within the surgical bed, whereas lesions were found at other anatomical sites (*p* < 0.001); moreover we found no evidence of a difference in the SUVmax according to anatomical site (*p* = 0.219).

**Table 3:** Distribution of avid [^18^F]PSMA-1007 uptake sites and characteristics (size and SUVmax) of associated lesions in *n* = 30 patients.

Anatomical site	Lesions (*n* = 149)	Size (mm)			SUVmax		
		Mean (SD)	Range	Intergroup comparison	Mean (SD)	Range	Intergroup comparison
Surgical bed, *n* (%)	18 (12.08%)	29.56 (15.69)	0–49	*p* < 0.001^c^^,^^d^	13.39 (14.07)	3.27–63.90	*p* < 0.219^c^
Lymph nodes, *n* (%)	62 (41.61%)	9.37 (6.78)	0–41		12.28 (12.46)	1.17–63.77	
Bone, *n* (%)	49 (32.89%)	10.29 (11.84)	0–50		12.71 (10.87)	1.97–61.16	
Loco-regional spread^a^, *n* (%)	14 (9.40%)	10.64 (13.75)	0–53		17.06 (16.25)	4.32–66.88	
Other sites^b^, *n* (%)	6 (4.03%)	3.00 (7.35)	0–18		8.66 (10.56)	1.79–30.00	

a Metastasis: avid uptake of [^18^F]PSMA in bladder (*n* = 9, 6.04%), seminal vesicle (*n* = 3, 2.01%) and rectum (*n* = 2, 1.34%).

b Metastasis: avid uptake for [^18^F]PSMA in thyroid (*n* = 4, 2.69%), kidney (*n* = 1, 0.67%) and bone marrow (*n* = 1, 0.67%).

c Kruskal-Wallis test.

d Dunn-Bonferroni post hoc test between Surgical bed group and each of the other four groups.

SD, Standard deviation.

The Spearman correlation coefficients (*r*) between lesion size (mm) and SUVmax for [^18^F]PSMA-1007 overall and grouped by lesion anatomical site are shown in Table 4. When considering all the lesions, a correlation was identified (*r* = 0.358, *p* < 0.001), but when grouping the lesions by anatomical site, a correlation was observed only for the lymph node lesions (*r* = 0.516, *p* < 0.001).

**Table 4:** Spearman’s correlation coefficients between size in mm and SUVmax for all lesions and grouped by anatomical site.

Size (mm)	SUVmax		
	*r*	IC 95%	Significance
Overall (*n* = 149)	0.358	(0.204, 0.494)	*p* < 0.001
Surgical bed (*n* = 18)	0.432	(−0.058, 0.755)	*p* = 0.073
Lymph nodes (*n* = 62)	0.516	(0.298, 0.682)	*p* < 0.001
Bone (*n* = 49)	0.242	(−0.051, 0.496)	*p* = 0.094
Loco-regional spread^a^ (*n* = 14)	0.232	(−0.356, 0.688)	*p* = 0.424
Other sites^b^ (*n* = 6)	0.655	(−0.364, 0.960)	*p* = 0.158

a Bladder (*n* = 9), seminal vesicle (*n* = 3) and rectum (*n* = 2).

b Thyroid (*n* = 4), kidney (*n* = 1) and bone marrow (*n* = 1).

Of the total number of lesions (*n* = 149) among the 30 patients, only those corresponding to 28 patients were classified with both the PSMA-RADS and the E-PSMA systems (*n* = 137); the E-PSMA was not used for two patients whose uptake levels in the spleen were higher than those in the parotid gland, making it impossible to determine the miPSMA expression of the associated lesions. Table 5 shows the frequency and percentage of each of the lesion classification categories based on the PSMA-RADS and the E-PSMA classification systems; 78/137 (56.93%) of the lesions were classified as PSMA-RADS-5, and 79/137 (57.66%) of the lesions were classified as E-PSMA-5. Additionally, substantial agreement was detected between the two systems (κ = 0.70, *p* < 0.001), but there was disagreement between the PSMA-RADS and E-PSMA classification systems in 24/137 (17.52%) of the lesions; of note, 18/137 (13.14%) were classified as PSMA-RADS grade 4 but E-PSMA grade 3.

**Table 5:** Contingency table for the classification of lesions (*n* = 137) among 28 patients according to the PSMA-RADS and E-PSMA systems*.

PSMA-RADS		E-PSMA					Total
		1	2	3	4	5	
1A	1	**2** ^**a**^	1	1	0	0	4 (2.92%)
1B							
2	2	0	**5** ^**a**^	0	0	0	5 (3.65%)
3A	3	0	0	**1** ^**a**^	1	0	2 (1.46%)
3B							
3C							
3D							
4	4	0	0	18	**28** ^**a**^	2	48 (35.04%)
5	5	0	0	0	1	**77** ^**a**^	78 (56.93%)
Total		2 (1.46%)	6 (4.38%)	20 (14.60%)	30 (21.90%)	79 (57.66%)	137 (100%)

a Concordant lesions between PSMA-RADS and E-PSMA systems.

*Kappa index (95% CI) = 0.70 (0.61–0.80), *p* value < 0.001.

We observed that in 5 of the 24 discordantly classified lesions, E-PSMA yielded a higher classification level than did PSMA-RADS. These cases included degenerative changes in the cervical vertebral body in one patient, a high-uptake bladder abscess in another patient, thickening of the rectum with high uptake in a third patient and a pair of nodes in the final patient with a short-axis diameter of 8 mm that were morphologically preserved yet demonstrated radiotracer uptake.

Among the other 19 of the 24 discordant lesions between the systems, PSMA-RADS produced a higher classification than did the E-PSMA system. These lesions had the following characteristics: a lymph node lesion with a short-axis diameter of 7 mm that demonstrated morphological changes and a miPSMA score of 3, and 18 lesions with a PSMA-RADS grade of 4 but an E-PSMA grade classified as 3, all of which lacked morphological changes with miPSMA uptake of 1 (that is, uptake between that of the blood pool and the spleen). Of these 18 lesions, seven were in lymph nodes with a short axis diameter ranging from 3 to 6 mm, seven were in bone, three were in the surgical bed and one was in the bladder.

## 4 Discussion

PSMA PET/CT could substantially impact the clinical management of PCa patients based on its diagnostic accuracy (23). Recent studies have used [^18^F]PSMA-1007 to detect PCa, identify the presence of metastases before treatment (24) and to determine BCR after treatment (25). Ongoing initiatives are underway to evaluate the reliability and practicality of standardized reporting systems for PSMA PET/CT scans in PCa patients (26), providing external validation. The use of lesion classification systems like PSMA-RADS and E-PSMA, which rely on criteria such as location, morphology, size, and radioisotope uptake (SUVmax), aims to mitigate potential confirmation bias. Nevertheless, incorporating the patient’s clinical history during the study may inadvertently lead to focusing on specific body systems or organs, potentially neglecting other areas. This approach could lead to the omission of small lesions, highlighting the critical importance of conducting a thorough and impartial evaluation of all potential abnormalities.

Our study expands the knowledge about the presence of metastases in patients with PCa post-prostatectomy, indicating that the largest metastatic PCa lesions, in terms of size, were found within the surgical bed when compared to lesions in other anatomical sites (*p* < 0.001). Regarding lymph node lesions, our study revealed a positive correlation (*r* = 0.516, *p* < 0.001) between the size of these lesions and their [^18^F]PSMA-1007 uptake. Furthermore, we identified substantial agreement between the PSMA-RADS and E-PSMA classification system scores among all lesions (κ = 0.70, *p* < 0.001), and the greatest disagreement between the two scores occurred mainly among lymph node lesions.

In cases where E-PSMA resulted in a higher classification level than PSMA-RADS for lesions, these differences can be attributed to the fact that the PSMA-RADS provides the flexibility to categorize lesions that are typically benign with and without uptake, apart from identifying likely malignant nonprostate lesions. Additionally, the PSMA-RADS system does not incorporate the criterion of an 8 mm size for lymph node lesions as proposed in the E-PSMA system. Regarding lesions with discordant classifications where PSMA-RADS assigned a higher classification than did E-PSMA, these disparities can be elucidated by considering that the E-PSMA system accounts for uptake levels based on reference values (miPSMA) and suggests a standardized size criterion of 8 mm for lymph node lesions.

Some points to consider regarding these discordances in lesion classification include the fact that the uptake of PSMA ligands has been reported in a variety of benign conditions associated with osteoblastic activity, including osteoarthritis, degenerative changes, fibrous dysplasia, consolidation fractures and post radiotherapy (27). In addition, bone changes on CT scans are frequently absent. The uptake of PSMA ligands may be associated with endothelial cell neovascularity as well as the high permeability of inflammatory cells (28). Moreover, Luo et al. (29) reported excellent performance in the detection of PCa using [^18^F]PSMA-1007 PET/CT, which was validated by histopathology. Additionally, they determined that an optimal SUVmax threshold of 8.3 could be applied to identify PCa lesions through [^18^F]PSMA-1007 PET/CT.

Regarding discordant lesions in the lymph nodes, recent studies, such as the study by Gottlieb et al. (30), revealed that histopathologically, the tumor burden in lymph node lesions is associated with BCR-free survival time in PCa patients. Furthermore, Schwartz et al. (31) described that the stratification of lymph node lesions based on size and the anatomical region can hinder the serial follow-up of the lesions and potentially provide discordant findings in their classification. However, there is controversy regarding whether the size of the short axis or the long axis should be measured. On the one hand, the Prostate Cancer Clinical Trials Working Group (32) proposed a long axis ≥ 20 mm as a strict criterion for a lesion in a metastatic lymph node in PCa; on the other hand, Hövels et al. (33) suggested that lesions in lymph nodes with a short axis > 8 mm in the pelvis and > 10 mm outside the pelvis can be considered malignant in PCa. Finally, Schwartz et al. (31) reported that when only the short axis of the lymph node lesion is considered, there is a better and more significant correlation with the tumor burden, and they recommend adopting the measurement of the short-axis diameter for lymph node lesions in radiological practice.

This study has certain limitations. First, this was a cross-sectional study, so causality cannot be inferred. Second, the results are based on data from a single center with a small sample size, and from both adult patients (*n* = 32) and lesions (*n* = 149) among a population with post-prostatectomy PCa and suspected recurrent disease; therefore, the results should be interpreted with caution. Third, histopathological confirmation of the lesions was lacking but we define recurrent disease (local recurrence, regional nodal involvement and distant metastases) based on BCR post prostatectomy (at least two prostate-specific antigen (PSA) values that are 0.2 ng/mL or higher).

In future perspectives, radiomics emerges as a promising tool for the detection and categorization of PCa lesions (34). Radiomic signatures, generated through the amalgamation of information from PET/CT and PSMA, hold the potential to provide complementary insights into the detection and localization of PCa lesions, as well as predicting PSMA-RADS or E-PSMA outcomes. To advance our understanding, it is necessary to carry out longitudinal multicenter studies that allow lesion follow-up and assessment of their response to treatment, especially in cases where lesions are discordant between the PSMA-RADS and E-PSMA classification systems, particularly with an emphasis on histopathological confirmation.

## 5 Conclusion

Our study expands the knowledge about the presence of recurrent disease (local recurrence, regional nodal involvement and distant metastases) based on BCR in patients with PCa and postprostatectomy, revealing that the largest lesions were located in the surgical bed, while the lesions with the highest uptake of [^18^F]PSMA-1007 were located in the lymph nodes. Regarding lymph node lesions, our study generates evidence of a positive correlation between the size of these lesions and [^18^F]PSMA-1007 uptake. Furthermore, we identified that although there was substantial agreement between the two lesion classification systems (PSMA-RADS and E-PSMA), there were discrepancies, mainly among the lymph node lesions. From clinical practice, our study suggests that lymph node lesions require special attention in their classification to ensure a correct diagnosis and for better decision-making regarding their respective management. Finally, it is necessary to carry out longitudinal multicenter studies that allow lesion follow-up and assessment of their response to treatment, including their histopathological confirmation, especially those lesions that were discordant between the PSMA-RADS and E-PSMA classification systems.

## Data availability statement

The raw data supporting the conclusions of this article will be made available by the authors, without undue reservation.

## Ethics statement

The studies involving humans were approved by Research and Research-Ethics committees of the HRAEB (registration numbers: CI-HRAEB 019-2023 and CEI-021-2023). The studies were conducted in accordance with the local legislation and institutional requirements. The ethics committee/institutional review board waived the requirement of written informed consent for participation from the participants or the participants’ legal guardians/next of kin given the retrospective nature of the study. The confidentiality of the data was meticulously maintained, and all procedures adhered to the pertinent guidelines and regulations governing this research.

## Author contributions

MM-Á: Conceptualization, Formal analysis, Methodology, Project administration, Software, Supervision, Validation, Writing – original draft, Writing – review & editing. HE-P: Conceptualization, Formal analysis, Methodology, Project administration, Supervision, Validation, Writing – original draft, Writing – review & editing. JC-L: Conceptualization, Data curation, Methodology, Writing – original draft, Writing – review & editing. ER-M: Conceptualization, Formal analysis, Methodology, Project administration, Software, Supervision, Validation, Writing – original draft, Writing – review & editing.
